# Stilbenes and resveratrol metabolites improve mitochondrial fatty acid oxidation defects in human fibroblasts

**DOI:** 10.1186/1750-1172-9-79

**Published:** 2014-06-05

**Authors:** Virginie Aires, Dominique Delmas, Carole Le Bachelier, Norbert Latruffe, Dimitri Schlemmer, Jean-François Benoist, Fatima Djouadi, Jean Bastin

**Affiliations:** 1 Laboratoire de Biochimie (Equipe Bio-peroxIL), Université de Bourgogne, Dijon, France; 2 INSERM UMR 866, Université de Bourgogne, Dijon, France; 3 INSERM UMR-S 1124, Université Paris Descartes, UFR Biomédicale des Saints-Pères, 45, rue des Saints-Pères, 75270 Paris cedex 06, France; 4 Assistance Publique Hôpitaux de Paris, Paris, France; 5 Hôpital Robert Debré, Centre de Référence des Maladies Héréditaires du Métabolisme, Service de Biochimie-Hormonologie, Paris, France

**Keywords:** Mitochondrial FAO defects, Resveratrol, Pharmacological therapy, Patient fibroblasts

## Abstract

**Background:**

Inborn enzyme defects of mitochondrial fatty acid beta-oxidation (FAO) form a large group of genetic disorders associated to variable clinical presentations ranging from life-threatening pediatric manifestations up to milder late onset phenotypes, including myopathy. Very few candidate drugs have been identified in this group of disorders. Resveratrol (RSV) is a natural polyphenol with anti-oxidant and anti-inflammatory effects, recently shown to have beneficial metabolic properties in mice models. Our study explores its possible effects on FAO and mitochondrial energy metabolism in human cells, which are still very little documented.

**Methods:**

Using cells from controls and from patients with Carnitine Palmitoyl Transferase 2 (CPT2) or Very Long Chain AcylCoA Dehydrogenase (VLCAD) deficiency we characterized the metabolic effects of RSV, RSV metabolites, and other stilbenes. We also focused on analysis of RSV uptake, and on the effects of low RSV concentrations, considering the limited bioavailability of RSV *in vivo*.

**Results:**

Time course of RSV accumulation in fibroblasts over 48 h of treatment were consistent with the resulting stimulation or correction of FAO capacities. At 48 h, half maximal and maximal FAO stimulations were respectively achieved for 37,5 microM (EC50) and 75 microM RSV, but we found that serum content of culture medium negatively modulated RSV uptake and FAO induction. Indeed, decreasing serum from 12% to 3% led to shift EC50 from 37,5 to 13 microM, and a 2.6-3.6-fold FAO stimulation was reached with 20 microM RSV at 3% serum, that was absent at 12% serum. Two other stilbenes often found associated with RSV, i.e. cis- RSV and piceid, also triggered significant FAO up-regulation. Resveratrol glucuro- or sulfo- conjugates had modest or no effects. In contrast, dihydro-RSV, one of the most abundant circulating RSV metabolites in human significantly stimulated FAO (1.3-2.3-fold).

**Conclusions:**

This study provides the first compared data on mitochondrial effects of resveratrol, its metabolites, and other natural compounds of the stilbene family in human cells. The results clearly indicate that several of these compounds can improve mitochondrial FAO capacities in human FAO-deficient cells.

## Background

Resveratrol (RSV) or trans-3, 4′, 5-trihydroxystilbene is a natural polyphenol of the stilbene family produced by plants in response to environmental stress, which is found in different fruits (red grape, berries, peanuts, etc.…) and in plant-based foods, in particular in red wine. RSV was initially shown to exhibit anti-oxidant, anti-inflammatory, and anti-proliferative effects in various cell systems, with potential applications in cancer and cardiovascular diseases [1-3].

More recent studies established that dietary supplementation with relatively high doses of RSV could provide resistance to obesity, and improve muscular performance, in mice fed a high-fat diet, whereas these effects were not observed at lower doses of RSV [4,5]. This raised the hypothesis that RSV could impact mitochondrial energy metabolism. In line with this, other data suggest that, at least in some metabolically compromised states, RSV supplementation might modulate mitochondrial functions in liver, skeletal muscle, or adipose tissue [6,7].

Chronic *in vivo* administration of RSV for long periods of time might variably affect many enzymes and metabolic pathways, through direct or indirect mechanisms. Accordingly, delineating the mitochondrial response to RSV requires *ex vivo* studies in cell models. There are yet limited data suggesting that treatment with RSV could impact mitochondrial fatty acid ß-oxidation [5,8-10] or oxidative phosphorylation in cultured cells [10]. These effects of RSV might find applications for the treatment of various diseases involving mitochondrial dysfunctions, such as diabetes, cardiovascular or neurodegenerative disorders [2].

Carnitine Palmitoyltransferase 2 (CPT2) and Very Long Chain AcylCoA Dehydrogenase (VLCAD) deficiencies belong to a group of more than fifteen genetic diseases affecting one of the enzymes of the mitochondrial ß-oxidation pathway, well characterized at the clinical and molecular levels, but for which treatments remain quite limited [11,12]. A wide panel of mutations is encountered in these disorders, and a majority of patients harbor missense mutations compatible with the production of unstable variant enzymes with very low residual activity. This results in a significant reduction in the capacity to use long-chain fatty acids as energy substrate, as typically observed in fibroblasts from patients with the myopathic form of CPT2 or VLCAD deficiency [13,14]. Clinically, these deficiencies translate into a limited tolerance to metabolic stress conditions such as exercise, fasting, or exposure to cold, which can trigger severe muscular manifestations in CPT2 or VLCAD-deficient patients [15]. In the past years, we explored therapeutic approaches aimed at stimulating the production of mutated enzyme [15,16]. Based on available literature data, RSV was the only dietary component eventually capable to induce stimulation of mitochondrial ß-oxidation, at least after chronic *in vivo* administration in mice. This led us to test the effects of RSV in ß-oxidation deficient fibroblasts, and these initial studies established that exposure to RSV (75 μM) could normalize FAO capacities in patients cells with the muscular form of CPT2 or VLCAD deficiency [17].

Data on the therapeutic potential of RSV in human are still limited, but it is now admitted that RSV could be used safely at a dosage ranging from a few milligrams up to 1 gram per day [3,18]. Following ingestion, trans-RSV, the most abundant natural RSV isomer, is quickly absorbed and metabolized, and converted into a variety of metabolites or conjugated forms, whose levels reach up to 20-fold those of the parent molecule [18-20]. This feeds the debate on the relevance of *ex vivo* observations performed in cells treated with high concentrations of trans-RSV [3,20,21]. On the other hand, the wide potential of this natural compound, its low price, and its excellent tolerance in humans, are strong incentives to further characterize its effects, especially for possible applications in diseases with few treatments to date, such as CPT2 or VLCAD deficiency.

When considering possible effects of RSV, both *in vivo* or at the cell level, a large number of parameters could be involved, many of which have little, or not, been characterized. For example, possible effects are primarily expected to depend on the uptake of RSV by various tissues or cell types, but the kinetics of RSV influx/efflux have been studied only in few instances [22,23]. Another important issue is to determine if some cell culture system components might affect RSV availability, and hence cellular response. Considering the limited bioavailability of RSV, it also appears essential to characterize the cell responsiveness to low, rather than to saturating, concentrations of RSV. Finally pharmacokinetics data raise questions about the role of RSV metabolites, which quickly accumulate after RSV ingestion, in mediating the effects of their parent molecule [24]. In particular, there are no data on possible effects of RSV metabolites on energy metabolism.

Accordingly, the first aim of the present study was to perform pharmacological characterization of RSV effects on FAO in cultured patient cells, with special focus on analysis of RSV uptake and on the metabolic effects of low RSV concentrations. In parallel, we analyzed possible mitochondrial effects of the main RSV metabolites identified in human, which had never been evaluated so far. Finally, we also tested metabolic effects of other naturally occurring stilbenes commonly found in association with RSV in plants and food.

## Methods

### Human fibroblasts and cell treatments

CPT2-deficient, VLCAD-deficient, or control human skin fibroblasts used in this study have been described previously [14,25]. Mutations and genotypes are given in Table 1. Under standard conditions, fibroblasts were grown in complete Ham’s F10 medium (Invitrogen) with glutamine, 12% fetal calf serum (FCS), 100 U/ml penicillin, and 0,1 mg/ml streptomycin. In some experiments the FCS was reduced to 3%. This low serum value was chosen because it allowed normal fibroblasts growth, in contrast to serum-free media that results in blocking cell proliferation. For treatment, the medium was removed and replaced with fresh medium containing the various compounds to be tested. These included RSV (Cayman Chemical company, USA), or one of the following compounds: cis-RSV (Cayman Chemical company, USA), piceid (Art Molecule, France), RSV-3-O-D-glucuronide (Art Molecule, France), RSV-4-O-D-glucuronide (Bertin Pharma, France), RSV-3-O-sulfate (Art Molecule, France), dihydroRSV (Art Molecule, France). Stock solutions of these compounds were prepared in DMSO and protected from light, and controls received equivalent amounts of DMSO (≤0.05%).

**Table 1:** Mutations of CPT2- or VLCAD-deficient patients

**Patients**	**Mutated gene**	**Nucleotides change**		**Amino acid change**	
1	CPT2	c.338C > T	c.371G > A	S113L	R124Q
2	CPT2	c.338C > T	c.112-113InsGC	S113L	S38Fs
3	VLCAD	c.1144A > C	c.1339G > A	K382Q	G447R
4	VLCAD	c.848 T > C	c.848 T > C	V283A	V283A
5	VLCAD	c.664G > A	c.1512G > T	G222R	E504D

The numbering of nucleotides starts at the first adenine of the translation initiation codon.

The numbering of amino acids starts at the first methionine encoded by the translation initiation codon.

### Fatty acid oxidation measurements

Fatty acid oxidation (FAO) was measured by the production of ^3^H_2_O from (9,10-^3^H) palmitate, as described previously [25]. Briefly, after removal of cell culture medium, fibroblasts seeded in 24-well plates were incubated in 200 μl/well of PBS buffer containing 125 μM tritiated palmitate + 1 mM carnitine, for 2 hours at 37°C. After incubation, the mixture was removed and de-proteinized, ^3^H_2_O was collected on ion-exchange resin, and the eluate was counted by liquid scintillation. The FAO values were expressed relative to protein content determined by the Lowry method.

### Acylcarnitine analysis

The method used has been described previously [26]. Briefly, fibroblasts were cultured in complete Ham’s F10 medium containing 200 μM palmitate and 400 μM carnitine for 72 hours at 37°C. The culture medium was collected, extracted, and analyzed for acylcarnitine content by electrospray MS-MS, using an API3000 triple quadrupole mass spectrometer (Sciex, Applied Biosystems, USA) detecting the precursors of an m/z ratio of 85, by reference to added internal deuterated standards. C16:0 acylcarnitines were quantified by reference to standard curves.

### Resveratrol uptake, efflux, and metabolism

Fibroblasts (0.7 × 10^5^) were seeded on 24-well plates in Ham’s F10 medium containing 12% FBS 24 h prior to treatment with [^3^H]RSV (specific activity: 74 GBq/mmol, Ge Health Care Life Science, Velizy-Villacoublay, France) at various concentrations and for several time points at 37°C. At the end of the incubation periods, the labeled medium was removed, and the cells were washed with PBS and lysed with NaOH 1 M. Cell-associated radioactivity was counted in a liquid scintillation analyzer (Perkin Elmer, Life Sciences Inc., Boston, MA). In parallel, cells treated with unlabeled RSV for the same kinetic time points, were used to measure protein content by the Lowry method.

For efflux experiments, cells were loaded with radiolabeled RSV (75 μM) for 1 h at 37°C (early maximal incorporation peak of RSV). After incubation, the labeled medium was removed and replaced by standard culture medium. Following this, the amount of RSV remaining in the cells was determined at various time points by counting cell-associated radioactivity.

For analysis of RSV metabolism, 10^6^ fibroblasts were treated by 75 μM RSV for 1 h and the medium was replaced by standard RSV-free medium. The presence of RSV aglycone and RSV metabolites was then analyzed in media and cell pellets at 0, 6 h, or 48 h after providing RSV-free medium, by LC-MS, (Agilent Technologies), as previously described [27].

### Western blot analysis

Western blots were performed as described previously [26]. The following antibodies were used: anti VLCAD (kindly provided by Dr S. Yamagushi, Japan), anti CPT2 (kindly provided by Dr C. Prip-Buus, France), anti porin (Abcam, Cambridge, UK), and anti ß-actin (Chemicon International, Temecula, USA). The bands were scanned by computerized densitometry (NIH Image J) and the results were expressed as arbitrary units, normalized to the amount of ß-actin.

### Statistical analysis

Data are the means ± SEM. Differences between groups were analyzed by one-way analysis of variance (ANOVA) and the Fisher test, or by the paired t test. P < 0.05 was considered significant.

## Results

### Exposure to trans-resveratrol can correct FAO deficiency in patient cells

**Figure 1:**
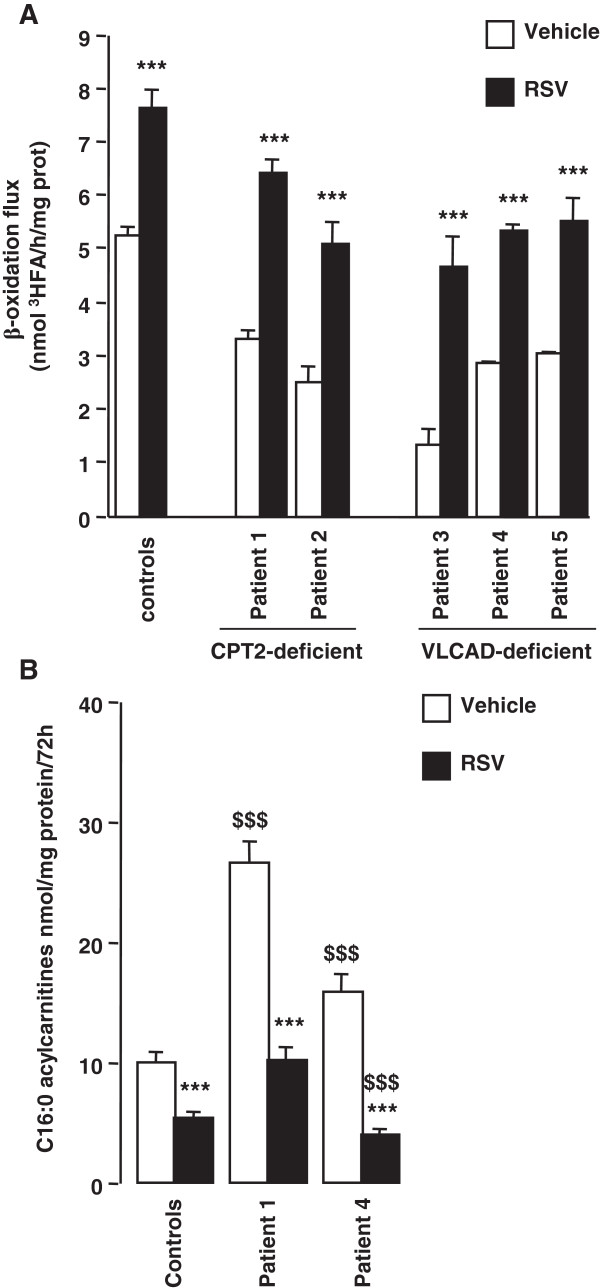
**Effects of 75 μM resveratrol for 48 h on ß-oxidation in fibroblasts from control, CPT2-, or VCAD-deficient patients. A)** Quantification of palmitate oxidation flux**.** The results are means ± SEM of at least three different experiments. In each experiment, the determinations were performed in triplicate. Control values are from three different healthy individuals. ***P < 0.001 compared with vehicle-treated fibroblasts. **B)** Quantification of C16:0 acylcarnitine production by tandem mass spectrometry. Results are expressed relative to cell proteins and are means ± SEM. ***P < 0.001, when compared with vehicle-treated cells, $$$ P < 0.001 compared with control fibroblasts.

As shown in Figure 1A, fibroblasts from patients with CPT2 or VLCAD deficiency typically exhibited markedly reduced palmitate oxidation capacities (from 1.3 to 3.3 nmol/h/mg), compared to control fibroblasts (5.2 ± 0.2 nmol/h/mg). Under standard culture conditions, treatment of fibroblasts by 75 μM RSV for two days led to maximal increases in palmitate oxidation in control (+45%) and patient cells (from +83 to +252%). This resulted in the restoration of normal FAO rates in all the CPT2- or VLCAD-deficient treated cells. The beneficial effect of RSV on FAO was further investigated by analysis of acylcarnitine profiles. As seen in Figure 1B, abnormal accumulation of long-chain acylcarnitines was observed in untreated CPT2 or VLCAD deficient fibroblasts, and a 3-day treatment with 75 μM RSV normalized the C16:0 acylcarnitine production.

### Time course of resveratrol uptake in cultured fibroblasts

**Figure 2:**
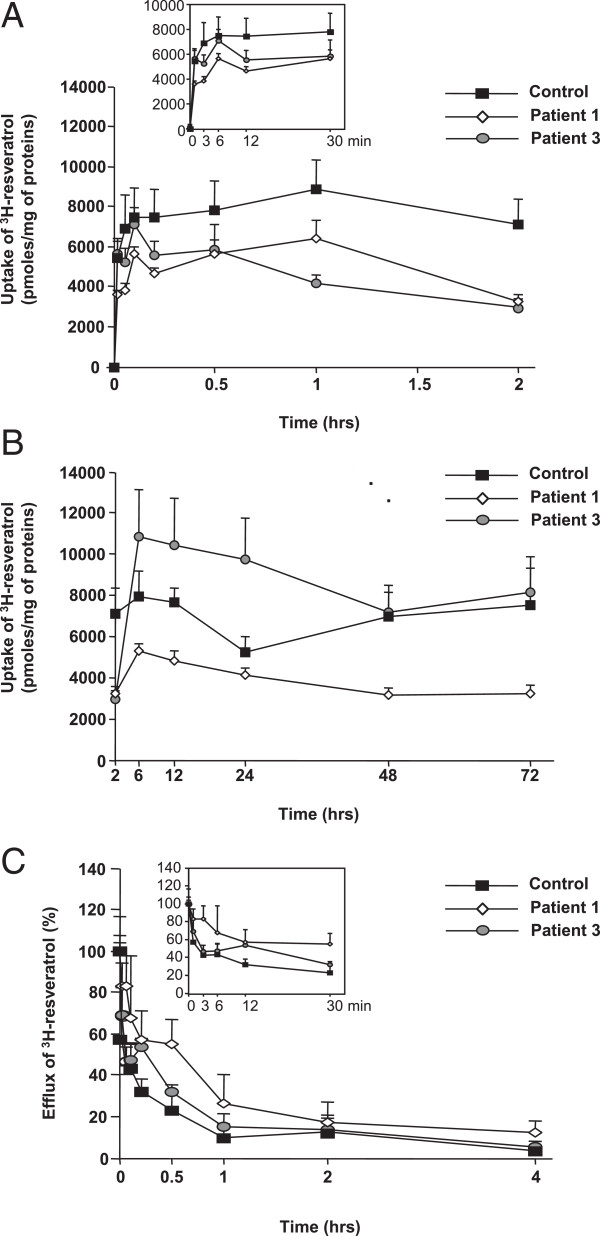
**Time-course of resveratrol uptake and efflux by control and CPT2 (patient 1)- or VLCAD (patient 3)-deficient fibroblasts. A)** and **B)** Cells were incubated at 37°C with tritiated RSV (75 μM) for the indicated times at 37°C. The uptake of labeled RSV was assessed as described in Methods. Data points are means ± SEM of at least three independent experiments each performed in triplicate wells. **C)** Cells were loaded for 1 h with tritiated RSV (75 μM, 37°C), the labeled medium was removed from the well and replaced by fresh unlabeled medium to measure the remaining cell-associated radioactivity, reflecting RSV efflux at the indicated time points. The values represent means ± SEM relative to uptake value observed after 1 h of treatment (maximal RSV uptake by cells). Data are from a representative experiment among two and each point represents the mean of three determinations on separate wells.

Under standard culture conditions, RSV (75 μM) was rapidly incorporated into control, CPT2- and VLCAD-deficient fibroblasts at 37°C, and its uptake was only discretely modified by low temperature (4°C; data not shown). In short-term experiments, uptake values in control cells reached about 90% of their maximal value at 12 minutes and remained stable up to 120 minutes, around 7000 pmol/mg proteins (Figure 2A). Interestingly, similar uptake levels were still observed after a 48 or 72 h exposure to RSV (Figure 2B). Patient 1 and patient 3 uptake values were in the range of 3000 to 6000 pmol/mg proteins between 12 and 120 minutes. In long-term experiments, values of RSV uptake in patient 1 generally remained lower than in control fibroblasts, In contrast, RSV accumulation in patient 3 fibroblasts transiently rose between 6 and 24 hours, and went back to control values at 48 and 72 hours. Thus, CPT2-deficient cells tended to incorporate less RSV than control or VLCAD-deficient cells (Figure 2B). However, RSV incorporation in long-term experiments remained at high levels in the three fibroblasts cell lines, representing more than 50% of their maximal uptake value (Figure 2B), and indicating a steady-state between RSV influx and efflux rates.

### Efflux and metabolism of resveratrol in control and patient fibroblasts

After exposure to 75 μM RSV for one hour, measurements of efflux showed that RSV was rapidly released in the extracellular medium, and 60% to 80% of intracellular RSV content was lost after 2 h (Figure 2C). In parallel, no detectable production of RSV glucuro- conjugates, and a very low level of RSV-3-O-sulfate, were measured (Table 2 and Table 3).

**Table 2:** Concentration of resveratrol and its metabolites in cell culture medium

	**Supernatant concentration (μM)**								
	**Control**			**Patient 1**			**Patient 3**		
**Species EFFLUX (hrs)**	**0**	**6**	**48**	**0**	**6**	**48**	**0**	**6**	**48**
***Resveratrol***	65.24 ± 7.14	5.49 ± 0.41	3.23 ± 0.58	61.07 ± 5.81	3.12 ± 0.81	3.09 ± 0.07	70.34 ± 0.73	4.20 ± 0.33	3.59 ± 0.74
***Resveratrol-3-0-sulfate***	0.02 ± 0.01	0.70 ± 0.09	4.01 ± 0.60	0.02 ± 0.004	0.40 ± 0.12	3.68 ± 0.04	0.02 ± 0.002	0.33 ± 0.03	2.37 ± 0.47
***Resveratrol-3-0-glucuronide***	nd.	nd.	nd.	nd.	nd.	nd.	nd.	nd.	nd.
***Resveratrol-4′-0-glucuronide***	nd.	nd.	nd.	nd.	nd.	nd.	nd.	nd.	nd.

**Table 3:** Concentration of resveratrol and its metabolites in fibroblasts

	**Cellular concentration (nmoles/mg of proteins)**								
	**Control**			**Patient 1**			**Patient 3**		
**Species EFFLUX (hrs)**	**0**	**6**	**48**	**0**	**6**	**48**	**0**	**6**	**48**
***Resveratrol***	14.96 ± 2.12	1.30 ± 0.21	0.83 ± 0.06	22.06 ± 1.95	1.29 ± 0.11	0.74 ± 0.06	20.58 ± 1.40	1.30 ± 0.17	0.89 ± 0.15
***Resveratrol-3-0-sulfate***	0.15 ± 0.04	0.18 ± 0.02	0.35 ± 0.03	0.18 ± 0.05	0.18 ± 0.02	0.27 ± 0.02	0.08 ± 0.01	0.10 ± 0.01	0.17 ± 0.02
***Resveratrol-3-0-glucuronide***	nd.	nd.	nd.	nd.	nd.	nd.	nd.	nd.	nd.
***Resveratrol-4′-0-glucuronide***	nd.	nd.	nd.	nd.	nd.	nd.	nd.	nd.	nd.

n.d., Not detected.

### Resveratrol uptake is negatively regulated by fetal calf serum

**Figure 3:**
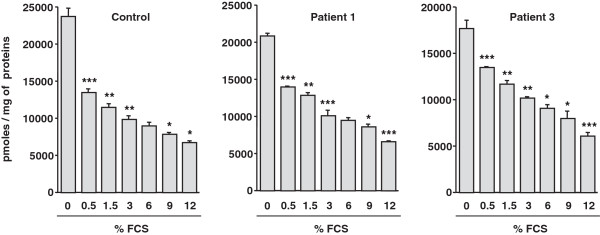
**Effect of fetal calf serum (FCS) on resveratrol uptake by control and CPT2- or VLCAD-deficient fibroblasts.** RSV uptake was examined by incubating control or patients’ fibroblasts with [^3^H]-RSV (75 μM) in serum-free medium or in medium supplemented with increasing FCS content for 1 h at 37°C. Data are means ± SEM of three determinations on separate wells. *P < 0.05, ***P < 0.001 vs. the previous column.

As shown in Figure 3, increasing fetal calf serum (FCS) content from 0.5 to 12% in the culture medium resulted in a marked decrease of RSV uptake values, both in control and in patient fibroblasts. This clearly suggests that some FCS components might reduce RSV bioavailability in the cell culture system.

### Changes in the response to resveratrol according to the FCS content of culture medium

**Figure 4:**
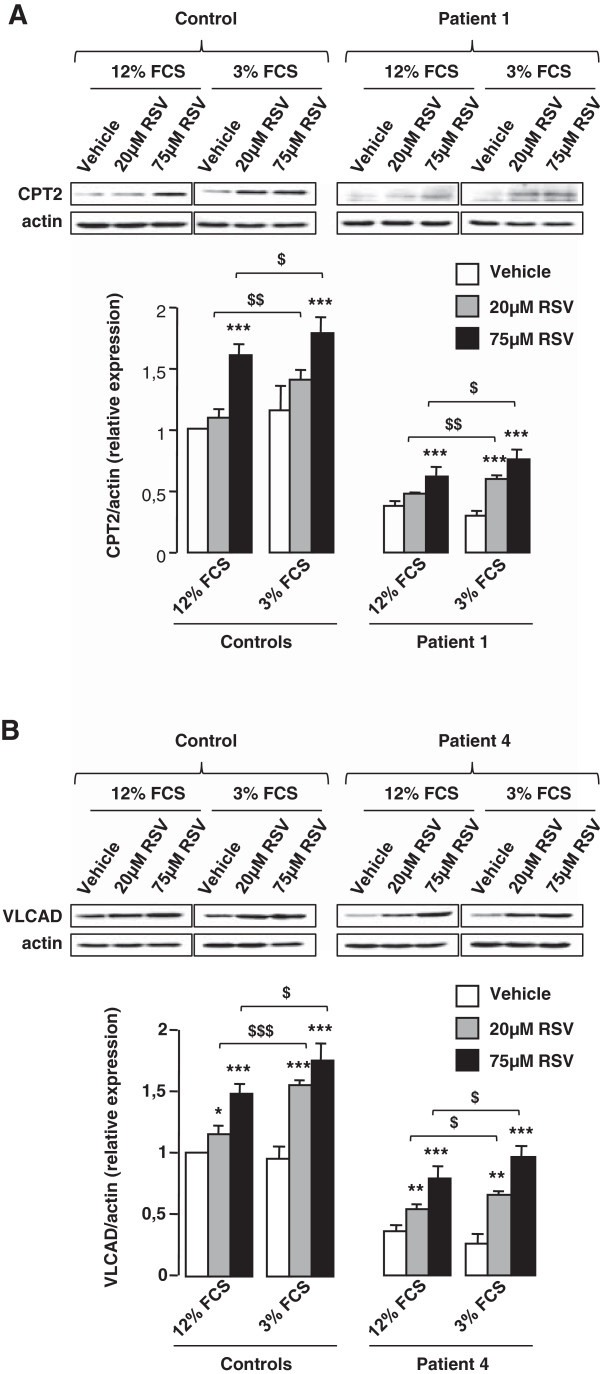
**Effects of medium FCS content on the response to RSV.** Cells were treated in 3% or 12% FCS medium with 20 or 75 μM RSV, or vehicle, for 48 h prior to protein extraction and western blot analysis. **A)** CPT2 protein levels in control or CPT2-deficient fibroblasts. **B)** VLCAD protein levels in control or VLCAD-deficient fibroblasts. Histograms of protein amounts normalized to ß-actin are shown, as well as representative western-blots. Values are means ± SEM of three westerns. Control values are from three different individuals. *P < 0.05, **P < 0.01, ***P < 0.001 when compared with vehicle-treated cells, $ P < 0.05, $$P < 0.01, $$$ P < 0.001 compared with cells treated in 12% FCS medium.

**Figure 5:**
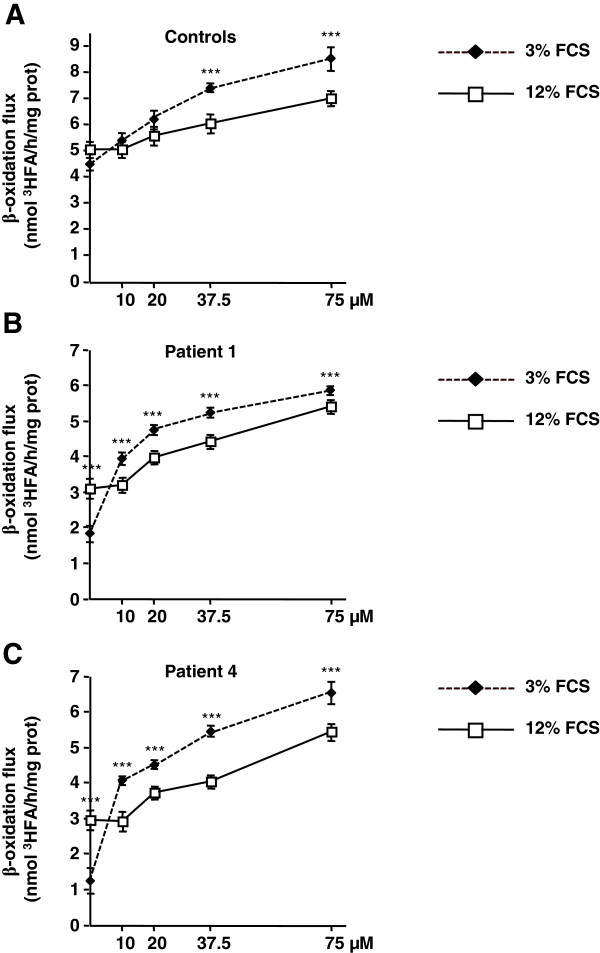
**Effects of resveratrol on FAO flux according to the FCS content in the culture medium, in control and FAO-deficient fibroblasts.** Dose–response studies of RSV effects on tritiated palmitate oxidation rates in **A)** controls **B)** CPT2- or **C)** VLCAD deficient patients’ fibroblasts. Cells were treated for 48 h. Control values are from three different individuals. The results are means ± SEM of at least three different experiments. In each experiment, the determinations were performed in triplicate. ***P < 0.001 when compared with cells treated in 12% FCS medium.

**Figure 6:**
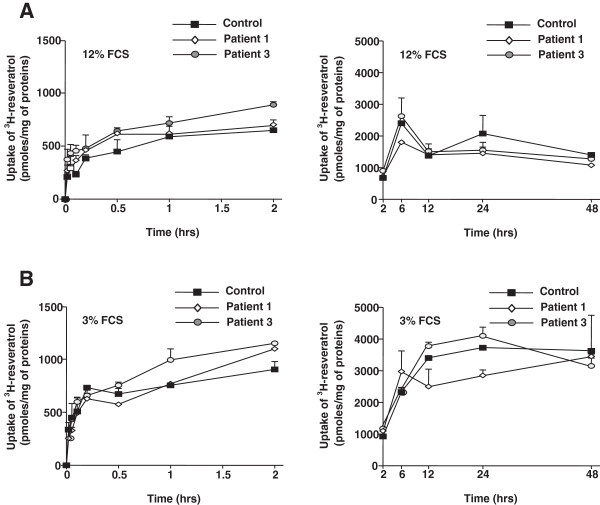
**Time-course of resveratrol (20 μM) uptake by control and FAO-deficient fibroblasts according to the FCS content in the culture medium.** RSV uptake was examined by incubating fibroblasts with [^3^H]-RSV (20 μM) in medium containing **A)** 12% or **B)** 3% FBS at 37°C for the indicated times. The values represent means ± SEM of two independent experiments each performed in triplicate wells.

We then compared the responses of control or patient cells to a 48 h treatment with 20 or 75 μM RSV in culture medium containing either 12% or 3% FCS. In the absence of RSV, western-blot experiments (Figure 4A and B) revealed a marked CPT2 or VLCAD protein deficiency in patient 1 and patient 4, respectively compared to control fibroblasts. Treatment with 75 μM RSV in 12% FCS medium resulted in a significant induction of CPT2 (Figure 4A) and VLCAD (Figure 4B) proteins in control and in patients’ cells. By contrast, treatment with 20 μM RSV in 12% FCS induced either modest increases or no changes in CPT2 (Figure 4A) and VLCAD (Figure 4B) proteins levels. Noticeably, for both RSV concentrations tested, the levels of CPT2 and VLCAD proteins in control and patient fibroblasts were significantly augmented when treatment was performed in 3% FCS medium.We then measured FAO capacities in control, and in patient fibroblasts exposed to 10, 20, 37.5, or 75 μM RSV in culture medium containing 12% or 3% FCS (Figure 5). Control cells treated in 12% FCS exhibited modest increases in FAO unless RSV reached 37.5 μM, when half-maximal FAO stimulation was observed (p < 0.01) (Figure 5A). When control fibroblasts were treated in 3% FCS medium, there was an increase in the slope of FAO dose–response curve, indicating an amplified response to RSV. Vehicle-treated patient cells exhibited more pronounced FAO deficiencies when grown in 3% FCS compared to 12% FCS medium (Figure 5B and C). After treatment with RSV in 3% FCS medium, patient cells exhibited a shift of dose–response curves towards higher values, compared to those observed in 12% FCS medium. Noticeably, in patient cells grown in 3% FCS medium, robust responses were already observed at 10 μM RSV, which triggered a +228% and +115% increase in FAO in VLCAD and CPT2-deficient fibroblasts, respectively, whereas, no response was observed in 12% FCS.We then compared incorporation levels of 20 μM RSV in 12% and 3% FCS medium (Figure 6A and B). Between 0 and 2 hours, control and patient cells accumulated larger amounts of RSV when treated in culture medium containing 3% (about 1000 pmoles/mg proteins), compared to 12% (about 750 pmol/mg proteins) FCS. At 24 and 48 hours, cellular uptake values reached 3000–4000 pmol/mg proteins in 3% FCS, i.e. more than twofold those observed in 12% FCS.

### Effect of resveratrol metabolites and of other stilbenes in FAO-deficient patient cells

**Figure 7:**
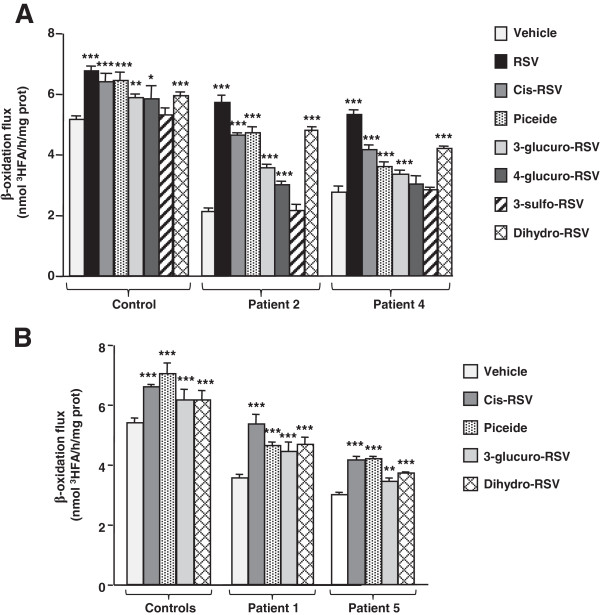
**Effects of natural stilbene compounds and RSV metabolites on FAO flux in control and patient fibroblasts.** Cells were treated with 75 μM of each compound for 48 h. **A)** compared effects of eight compounds in controls, CPT2- or VLCAD-deficient fibroblasts. **B)** compared effects of the most effective compounds in controls, CPT2- or VLCAD-deficient fibroblasts. Histograms are means ± SEM of at least three different experiments. Control values are from three different individuals. ***P < 0.001 compared with vehicle-treated fibroblasts.

**Figure 8:**
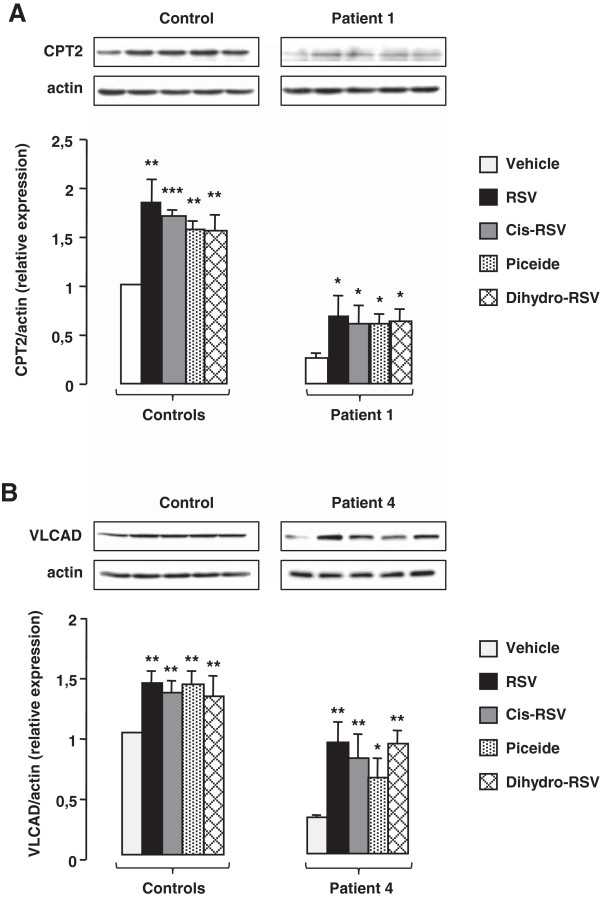
**Effects of natural stilbene compounds and RSV metabolites in control and patient fibroblasts.** Cells were treated with 75 μM of each compound for 48 h. **A)** changes in CPT2 protein levels in control and CPT2-deficient fibroblasts. **B)** changes in VLCAD protein levels in control and VLCAD-deficient fibroblasts. Histograms are means ± SEM of three different experiments. Control values are from three different individuals. *P < 0.05, **P < 0.01, ***P < 0.001 when compared with vehicle-treated cells.

We evaluated the effects of compounds structurally related to RSV, and of RSV metabolites. Two of them are stilbene compounds naturally found in association with RSV in foods, namely cis-RSV and trans-piceid [28]. The four others are major human metabolites i.e. RSV-3-O- and −4-O-glucuronide, RSV-3-O-sulfate, and dihydro-RSV. Cells were treated with 75 μM of each compound in 12% FCS medium for 48 h. Initial screening was performed in one control, one CPT2-deficient and one VLCAD-deficient cell line (Figure 7A). In control fibroblasts, exposure to cis-RSV or to piceid induced similar changes (+25%) in FAO. Treatment with these compounds resulted in a more pronounced increase in FAO in CPT2- (+121 to +125%, respectively) and in VLCAD-deficient fibroblasts (+30 to +50%, respectively). Treatment with each of four RSV metabolites tested generally resulted in modest changes (<15%) in FAO capacities in control fibroblasts. In patient cells, exposure to 3- or 4-glucuro-RSV led to moderate or no increase in FAO. In contrast, treatment with dihydro-RSV augmented FAO by 130 or 52% in CPT2 or VLCAD-deficient fibroblasts, respectively. The effects of these compounds were further analyzed in additional control, and deficient fibroblasts (Figure 7B). Altogether, these experiments established that exposure to cis-RSV, to piceid, or to dihydro-RSV triggered significant increases in FAO in control and patient cells. Consistent with this, cis-RSV, piceid, or dihydro-RSV induced a significant up-regulation of CPT2 (Figure 8A) and VLCAD (Figure 8B) proteins levels, accounting for the stimulatory effects of these compounds on FAO capacities.

### Resveratrol metabolites and other stilbenes induce mitochondrial biogenesis

**Figure 9:**
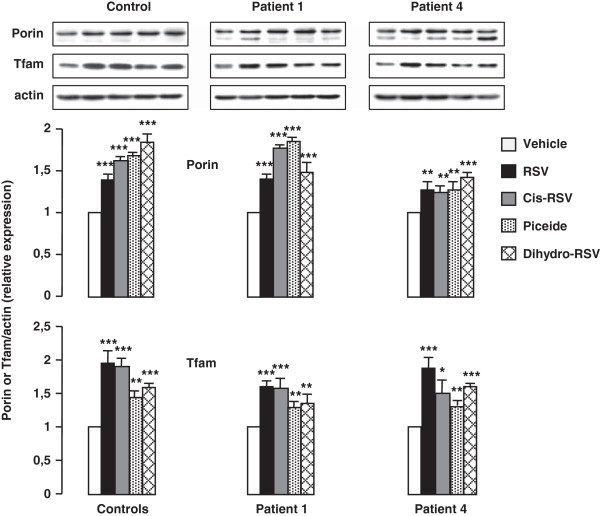
**Effects of natural stilbene compounds and RSV metabolites on porin and Tfam protein levels in control and patient fibroblasts.** Cells were treated with 75 μM of each compound for 48 h. Histograms are means ± SEM of three different experiments. Control values are from three different individuals. *P < 0.05, **P < 0.01, ***P < 0.001 when compared with vehicle-treated cells.

The effects of RSV, cis-RSV, piceid and dihydro-RSV on mitochondrial biogenesis was assessed by western blot analysis of two mitochondrial proteins generally admitted to reflect mitochondrial mass. Porin is a voltage dependent anion selective channel located in the outer mitochondrial membrane and Tfam, is the mitochondrial transcription factor A, which co-localizes with mtDNA in the mitochondrial nucleoids [29]. Western-blot analysis revealed a general stimulation of porin and Tfam protein expression in response to RSV, cis-RSV, piceid and dihydro-RSV, i.e. to all the compounds that were found to induce increases of FAO capacities (Figure 9).

## Discussion

The present study shows that exposure of CPT2- or VLCAD-deficient fibroblasts to RSV could induce up-regulation of mutated enzyme level, possibly leading to restore normal FAO capacities. This is in line with previous studies [17], and with other data suggesting that FAO is one of the main mitochondrial pathways impacted by RSV [5,8]. To gain further insights into its metabolic effects, we characterized the kinetics of RSV uptake. Control and FAO-deficient fibroblasts rapidly accumulated RSV, and, despite variability in the kinetics of RSV uptake between the cell lines, generally maintained high intracellular levels when chronically exposed to RSV. RSV uptake was modestly affected by low temperature suggesting that incorporation mainly proceeded via passive diffusion. In hepatoma cells, RSV accumulates both by passive diffusion and by carrier-mediated processes, however its content rapidly decreases after reaching its maximal value [23], in part because hepatoma cells metabolize RSV [30]. In contrast, our results indicate almost no detectable production of RSV metabolites by human fibroblasts, consistent with literature data showing that xenobiotic metabolism is extremely low in human fibroblasts [31-34].

Despite the large number of studies dealing with RSV effects in numerous cell types, there are few data on intrinsic factors that could affect the response to RSV in cultured cells. In this regards, our results show that intracellular RSV levels were significantly higher after treatment in 3% versus 12% FCS medium. In line with this, induction of CPT2 and VLCAD proteins, and stimulation of FAO capacities by RSV were markedly enhanced using low FCS medium. Thus, RSV uptake, and hence cell response to RSV, appeared negatively regulated by some FCS components. In our cultured fibroblasts, we found that addition of bovine serum albumin in serum-free culture medium already induced a marked dose-dependent decrease in RSV uptake (data not shown). Accordingly, negative effects of FCS on RSV uptake might be partly attributed to the high serum albumin content in the FCS. In line with this, some data suggest that albumin could behave as a RSV binding protein [22,35]. Overall, these observations might account for the high RSV concentrations commonly needed to trigger metabolic responses in cultured cells. Last, *in vivo* data indicate that a significant fraction of plasma RSV is bound to human serum albumin [19,20,36]. This protein-bound circulating RSV might serve as a reservoir to enhance *in vivo* half-life of this compound, by limiting its absorption and metabolism [36].

Exposure to RSV was reported to enhance FAO capacities in 3 T3 adipocyte [8] and in C2C12 muscle cells [5,9]. RSV might also stimulate mitochondrial ß-oxidation *in vivo*, as shown in a rat model of heart failure [37], or in rats submitted to endurance training [6]. Furthermore, a recent clinical trial in obese men receiving low doses of RSV (150 mg/day) established beneficial effects on energy metabolism, and suggested a preferred use of fatty acids as energy substrates under treatment by RSV [38].

The natural sources of RSV often associate a variety of other stilbenes. Thus, trans-piceid and cis-RSV are among the most abundant stilbenes present in foods, together with trans-RSV [28]. However, there were no data on possible effects of these compounds on energy metabolism. Interestingly, the present study indicates that cell exposure to cis-RSV, often considered as a by-product of trans-RSV, or to piceid, which represents the major form of RSV in plants, triggered a significant increase in ß-oxidation capacities in control and patient fibroblasts. Following ingestion in human, RSV is metabolized into glucuro- or sulfo-conjugates in the liver, and converted by intestinal microbiota into di-hydro RSV, one of the most abundant circulating RSV metabolites [18-20]. However, possible effects of these compounds on mitochondrial functions had never been investigated. Our results indicate that the 3-glucuronide derivative exhibited low stimulatory effects on FAO, whereas the 4-glucuro and 3-sulfo were ineffective. In contrast, dihydro-RSV appeared as a potent inducer of FAO capacities in patient fibroblasts. Overall, FAO assays in patient fibroblasts provide a sensitive approach to screen compounds potentially capable to induce stimulation of ß-oxidation in human cells [16]. The present study identifies several compounds that had not yet been described as regulating FAO capacities, including two natural stilbenes often associated to trans-RSV in food, i.e. cis-RSV and piceid [28] and one RSV metabolite i.e. dihydro-RSV. Interestingly, consumption of foodstuffs naturally rich in RSV will often lead to supply the three compounds to the organism, in addition to RSV.

From a mechanistic point of view, several studies indicate that RSV might stimulate mitochondrial biogenesis both *in vivo*, in mice submitted to high fat diet [5] or exercise [39], and *in vitro* in various cells [4,40]. In this study, we show that RSV and other related compounds triggered a stimulation of mitochondrial biogenesis that might underlie the increases in ß-oxidation flux. This is in line with our previous studies in human fibroblasts showing that RSV enhanced the protein level of PGC-1α, a master regulator of mitochondrial biogenesis [17,41].

## Conclusions

Altogether, our data show that human fibroblasts in primary culture stably incorporate RSV over a 48 h period, and do not produce significant levels of resveratrol metabolites. Exposure to RSV induced a marked increase in mitochondrial ß-oxidation flux, leading to the correction of FAO capacities in patients’ cells with the myopathic form of VLCAD or CPT2 deficiency. Pharmacological restoration of FAO by RSV was clearly associated with increased expression of mutant CPT2 or VLCAD proteins, as previously observed using bezafibrate. Increasing the level of misfolded proteins might theoretically induce secondary toxic effects, and in particular cellular oxidative stress [42]. In this regards, RSV, a known anti-oxidant compound, might have dual beneficial effects, possibly restoring FAO while counteracting oxidative stress in the treated cells. This study also brings the first data on mitochondrial effects of other natural compounds of the stilbene family, such as piceid and cis-RSV, which were both found to act as activators of mitochondrial FAO in control and in FAO-deficient fibroblasts. Similar approaches allowed screening RSV metabolites for their mitochondrial effects, and revealed that dihydro-RSV, one of the most abundant circulating resveratrol metabolites in human, could trigger a marked stimulation of FAO in deficient cells. Accordingly, this study suggests that RSV, in combination with its metabolites and with other naturally occurring stilbenes, might positively impact mitochondrial functions in human cells, possibly leading to the correction of FAO capacities in deficient cells. Accordingly, RSV and some related molecules might, at least in some cases, evolve from the status of micronutrients to that of natural drugs. It should be kept in mind, however that results from pre-clinical studies such as those obtained in the present study cannot be extrapolated to the *in vivo* situation, and will ultimately require to be tested in clinical trials in order to really evaluate the potential of RSV in these rare diseases.

## Abbreviations

FAO: Mitochondrial fatty acid ß-oxidation; RSV: Trans-resveratrol; CPT2: Carnitine palmitoyl transferase 2; VLCAD: Very long chain acylcoa dehydrogenase; FCS: Fetal calf serum; BSA: Bovine serum albumin; PGC-1α: Peroxisome proliferator-activated receptor gamma coactivator-1 alpha.

## Competing interests

The authors declare that they have no competing interests

## Authors’ contributions

JB and FD wrote the manuscript, and VA, NL, DD contributed to the writing. VA and CLB performed experiments. JB, FD, VA, NL, and DD analyzed the data. DS and JFB performed the acylcarnitine analysis. All authors read and approved the final manuscript.
